# Collateral circulation in a patient with combined traumatic radial and ulnar artery injuries

**DOI:** 10.1016/j.jvscit.2024.101583

**Published:** 2024-08-27

**Authors:** Sahil Patel, Samantha Klein, Tripti Mathur, Christopher Ingraham, Camille Jackson, Shahram Aarabi

**Affiliations:** a Department of Surgery, University of California San Francisco – East Bay, Oakland, CA; b University of California Berkeley, Berkeley, CA; c Department of Radiology, University of California San Diego, San Diego, CA

**Keywords:** Vascular surgery, Amputation, Collateral circulation, Hand anatomy, Abberant anatomy, Limb salvage

## Abstract

Combined radial and ulnar artery injuries are associated with a >35% amputation rate when not revascularized promptly. We describe a patient who suffered a penetrating injury to both the radial and ulnar arteries. Despite the ulnar artery being ligated and the radial artery primary repair becoming occluded after the index operation, his hand remained salvageable, likely because of collateral arterial pathways. The patient obtained a delayed radial-radial artery bypass and afterward achieved meaningful neuromotor function. This case emphasizes the importance of forearm vascular anatomy variability and the need for prompt management of arterial injuries to reduce limb loss and disability.

Primary arterial circulation to the hand comes from the superficial and deep palmar arches, arising from the radial and ulnar arteries.^1^ Injury to both radial and ulnar arteries is noted in 14% of penetrating injuries at the distal forearm.^2^ In a retrospective analysis of 77 patients, the amputation rate was 39.3% when both arteries were ligated for damage control vs <5% if only one artery was ligated.^3^ We present the case of a young man with an injury to both the radial and ulnar arteries, whose hand remained viable from collateral arterial pathways, despite delayed repair. This patient has agreed to have their case details and images published.

## Case report

A 31-year-old left-handed unemployed man was assaulted with a machete, sustaining multiple deep lacerations to his left volar wrist. On initial assessment, the patient had an unreliable motor and sensory exam with a Mangled Extremity Severity Score of 5. On emergent exploration of his left wrist, arterial bleeding from the ulnar artery was controlled via suture ligation. The distal aspect of the ulnar artery was not identifiable clearly in the wrist, so primary repair was not attempted. The transected radial artery was therefore revascularized because of signs of ischemia to the hand—dusky without capillary refill and no palmar arch Doppler signal. An end-to-end repair with 6-0 Prolene was performed, and multiphasic Doppler signals were noted proximal and distal to the anastomosis. No nerves were identified clearly during the index operation, and fasciotomies were not completed. The patient recovered in the surgical intensive care unit on a therapeutic heparin drip.

**Fig 1:**
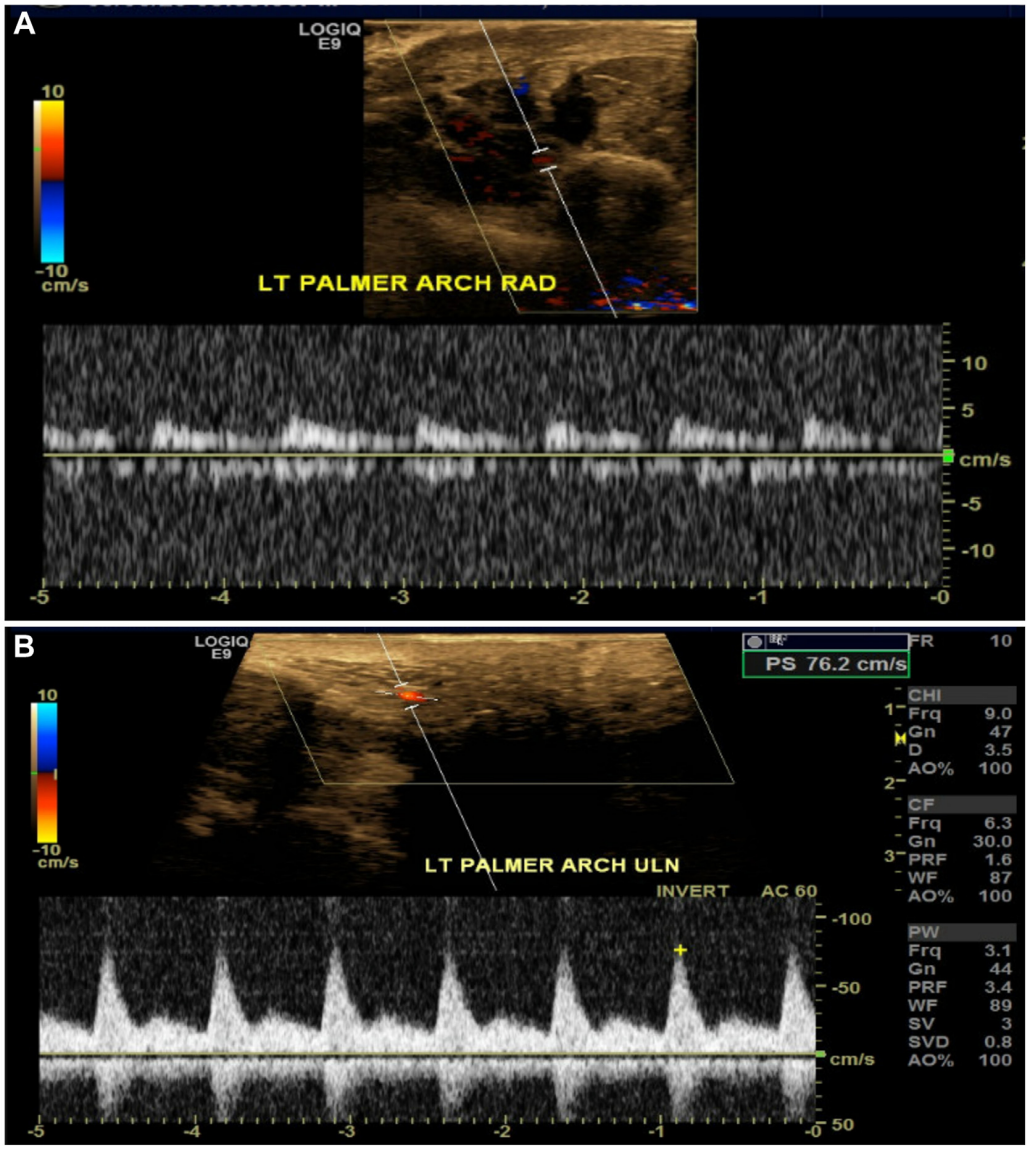
**(A)** Duplex arterial ultrasound image of the radial side of the palmar arch. Waveform exhibited is monophasic, intermediate resistive. **(B)** Duplex arterial ultrasound image of the ulnar side of the palmar arch. Waveform exhibited is multiphasic.

**Fig 2:**
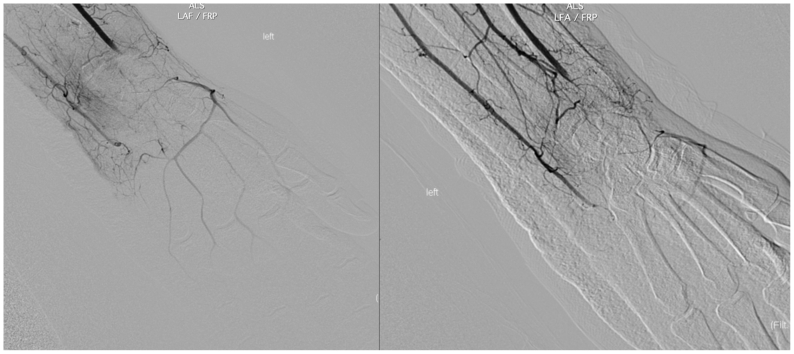
Diagnostic angiography after index operation. These images are taken shortly after contrast administration and show the radial and ulnar artery interrupted at the level of the wrist.

**Fig 3:**
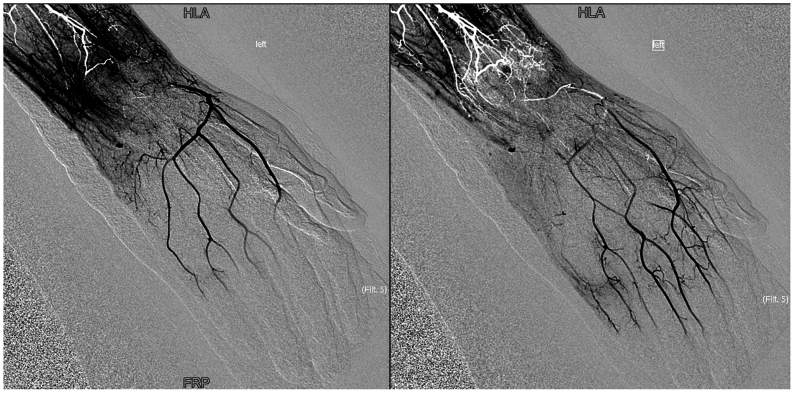
Diagnostic angiography after index operation. These images show intact palmar arch with delayed arterial flow, although the radial and ulnar artery are interrupted at the level of the wrist. Note the artifact (*white*) owing to stagnant flow in the distal wrist branches.

Day 1 postoperatively, computed tomography imaging showed multiple displaced carpal fractures and occluded distal radial and ulnar arteries immediately proximal to the wrist. Despite this finding, arterial duplex detected flow on the ulnar and radial sides of the palmar arch (Figs 1, *A* and *B*). Digit pressures on physiological noninvasive testing were 0 mm Hg in all five digits of the left hand. The patient could move his left digits, but noted decreased sensation to his hand. Capillary refill to the hand was delayed but present. No immediate revascularization attempts were made because neither a specialist nor transfer were available and the hand remained viable, without skin changes or worsening neuromotor deficits. A diagnostic angiogram showed the ulnar and radial arteries were interrupted at the level of the wrist, though arterial blood flow to the hand filled via multiple collaterals with an approximate 6- to 7-second delay (Fig 2, Fig 3 and Fig 2, Fig 3). Given the patient's stable neuromotor examination and imaging supporting delayed arterial flow into the hand, semielective repair with vascular surgery and hand surgery was planned.

Five days after the index operation, the patient returned to the operating room and the prior end-to-end radial artery repair was confirmed to be occluded. An incision parallel to the extensor pollicis longis tendon exposed the distal radial artery and a reversed greater saphenous vein graft was tunneled anatomically to perform a left radial artery (distal forearm) to radial artery (snuff box) bypass. Care was taken not to injure the superficial branch of the radial nerve. Multiphasic signals were obtained through the bypass graft and distal to the anastomosis. The hand surgery team repaired the flexor digitorum profundus and flexor digitorum superficialis of the second to fifth digits. The ulnar and median nerves were transected completely at the level of the wrist, so they were repaired primarily using microsurgical techniques. The patient recovered well and 3 weeks after this second operation, arterial duplex examination of the left extremity showed a patent radial-radial bypass graft. Digit-brachial indices were normal in all digits (>0.88). The patient has remained off therapeutic anticoagulation or antiplatelet agents after his bypass.

Over the 5 months after his second operation, the patient developed tendinosis of all joints of all his left-sided fingers that is being addressed with physical/occupational therapy. Sensation in the left hand continues to be diminished, but he reported using his left hand to complete basic activities of daily living.

## Discussion

Either the radial artery or ulnar artery needs to be patent distal to the elbow to maintain hand viability.^4^ Without prompt revascularization, the amputation rate is >35% if both these arteries are injured.^5^, ^6^, ^7^ According to case reports, limb viability can be maintained occasionally, even when both the radial and ulnar arteries are ligated, likely from aberrant vascular anatomy or collateralized blood flow.^5^^,^^8^, ^9^, ^10^

Typically, the brachial artery divides into the radial and ulnar artery at the level of the radial head. Accessory radial artery, persistent median artery (8.0%-27.1%), and other unnamed anomalous branching have been described.^10^ A complete superficial palmar arch is found with 90% frequency and is formed five ways: (1) by the superficial volar branch of the radial artery to the ulnar artery (40%); (2) entirely by the ulnar artery (35%); (3) by the ulnar and median arteries (approximately 15%); (4) by an anastomosis of the ulnar, median, and radial arteries (6%); and (5) by a branch of the deep palmar arch (4%). There are three typical deep palmar arch variations, formed by (1) the deep volar branch of the radial artery and the inferior deep branch of the ulnar artery (60%), (2) the deep volar branch of the radial artery and the superior deep branch of the ulnar artery (30%), and (3) an anastomosis of the deep volar branch of the radial artery with both deep branch of the ulnar artery (10%).^11^

In our patient, the ulnar artery was ligated, and the radial artery primary anastomosis occluded after the index operation. Viability of the left hand was likely due to collateral flow within the hand, observed during the diagnostic angiogram after the index operation. A later computed tomography scan showed that the ulnar artery divided into multiple branches from the mid to distal forearm, which may have allowed for sufficient blood flow to supply to the hand. Alternatively, the anterior interosseous artery may have been providing collateral flow, because angiography demonstrated that this artery was prominent at the level of the forearm. Although imaging showed no clear source to account for the blood flow through the palmar arch, the patient's hand remained viable through collateral blood flow.

Morbidity of penetrating injuries of the upper extremity is closely associated with damage to accompanying nerves, tendons, and bone fractures.^12^ Between 60% and 85% of patients requiring radial and/or ulnar artery repair after trauma have a concurrent nerve injury, and 40% to 81% have concurrent soft tissue injuries (tendon, ligament, or muscle).^13^, ^14^, ^15^ Current studies with small sample sizes indicate approximately 80% to 90% of patients recover function of the hand when nerve and soft tissue injuries are repaired in conjunction with arterial repair.^13^^,^^16^ In our patient, the hand was salvaged, despite transection of both major arteries and nerves at the level of the wrist. Ongoing hand functional deficits are likely owing to his ulnar and median nerve injury along with the tendonous damage he sustained.

## Conclusions

Given the high risk of hand loss, combined radial and ulnar artery injuries require immediate revascularization. In-line arterial flow through either the radial or ulnar arteries, as well as nerve exploration, should be performed at the index operation for these injuries. Our case illustrates that, despite delayed revascularization and repair of the major nerves and tendons, the hand could be salvaged with meaningful neuromotor function, likely owing to a nonstandard collateral circulation pathway.

## Disclosures

None.
